# Stress management training for managers in small and medium-sized enterprises (KMU-GO): results of a randomized controlled trial

**DOI:** 10.1186/s12889-025-26088-4

**Published:** 2026-01-13

**Authors:** Sophie Hofmann, Svenja Schlachter, Michael Gast, Rebecca Erschens, Carla Schröpel, Mathias Diebig, Susan Gritzka, Janina A. M. Lehmann, Elena Schwarz, Marc N. Jarczok, Elisabeth M. Balint, Urs M. Nater, Nadine Skoluda, Florian Junne, Peter Angerer, Harald Gündel

**Affiliations:** 1 Department of Psychosomatic Medicine and Psychotherapy, Ulm University Medical Center, Albert-Einstein-Allee 23, Ulm, 89081 Germany; 2 Department of Psychiatry and Psychotherapy II, Section Public Mental Health, Ulm University and BKH Günzburg, Ulm, Günzburg, Germany; 3 Department of Psychosomatic Medicine and Psychotherapy, Internal Medicine, University Medical Hospital Tübingen, Tübingen, Germany; 4 Work and Organizational Psychology, Trier University, Trier, Germany; 5 Institute of Occupational, Social and Environmental Medicine, Center for Health and Society, Faculty of Medicine, Heinrich-Heine-University Düsseldorf, Düsseldorf, Germany; 6 Center for mental health, Privatklinik Meiringen, University of Bern, Meiringen, Switzerland; 7 Department of Psychology, Institute of Clinical and Health Psychology, University of Vienna, Vienna, Austria; 8 Department of Psychosomatic Medicine and Psychotherapy, Otto von Guericke University Magdeburg, University Hospital Magdeburg, Magdeburg, Germany

**Keywords:** Stress prevention, Stress management training, Managers, Leaders, Small and medium-sized enterprises (SME), Mental health, Well-being, Randomized controlled trial

## Abstract

**Background:**

Leadership in small and medium-sized enterprises (SMEs) is associated with a variety of challenges and stressors, which are reflected in their managers’ commonly experienced high stress levels. In this context, psychological strain can arise, but compared to large companies, SMEs face particular difficulties in implementing mental health promotion intervention. These difficulties partially originate in a profound lack of comprehensive research on the effectiveness and prospects of success of intervention strategies in SMEs. This study aims to close this gap by evaluating a stress management training for managers in SMEs (KMU-GO). Implementing established stress management strategies and making them more accessible within the SME context, the training aims to improve participants’ psychological and physiological well-being.

**Methods:**

We conducted a randomized controlled trial with an intervention and a waitlist control group in Germany. The intervention comprised 1.5 days of stress management training and two refresher sessions, focusing on short- and long-term coping strategies and self-reflection. Based on a sample of *N* = 155 managers, we conducted a multilevel analysis of covariance on psychological measures, namely stress reactivity, depression, and anxiety. Additionally, changes in physiological stress indices (i.e., hair cortisol concentration and salivary alpha-amylase activity) were examined.

**Results:**

Regarding the psychological measures, we found significant training effects six months after baseline. Twelve months after baseline, there were no significant differences between the intervention and control groups for either psychological or physiological variables.

**Conclusion:**

In conclusion, the present stress management training is effective in reducing psychological strain in SME managers. In order to achieve more sustainable effects, a continuation of the refresher sessions could be considered.

**Trial registration:**

Before including the first participant, the KMU-GO trial is registered at the German Clinical Trial Register (DRKS): DRKS00023457 (registered on 5 November 2020).

## Background

Mental health conditions are widespread, contributing substantially to the global disease burden and causing significant economic costs [1–3]. For businesses, the considerable number of absenteeism days attributable to mental health conditions and the associated costs are highly relevant [4–6]. Work, as an important part of employees’ lives, can make a significant contribution to mental health, both positively and negatively. With regard to the latter, experiencing high levels of work-related stressors (e.g., high demands, role stress, perceived injustice) has been suggested as major detrimental contributor to employees’ mental health (e.g., [7, 8]). This is particularly evident when it comes to a mismatch between work demands and rewards [9, 10], which leads to systemic dysregulation due to prolonged stress [11, 12]. This dysregulation results from the cumulative physiological burden of chronic stress exposure, ultimately increasing the risk of developing various mental health conditions [13]. Consequently, promoting mental health in the workplace by addressing work-related stress is highly relevant for companies.

In small and medium-sized enterprises (i.e., enterprises with up to 500 employees; SMEs), mental health promotion is particularly crucial, as the impact of mental health conditions are severe and addressing them is more challenging compared to large companies [14]. Limited financial and structural resources make it difficult to manage the work loss and daily operations are more directly affected [14]. As SMEs often rely on a small group of workers, absenteeism can have a significant and more direct impact on the workload of other employees. Furthermore, replacing an absent employee may be challenging, and the loss of organizational knowledge may cause significant problems, as in SMEs, a single employee may exclusively represent an area of responsibility [5]. Given that in Germany, similar to other industrialized countries, 99.3% of companies are defined as SMEs, employing 56% of the workforce, this type of company is a highly pivotal target group for mental health promotion [15]. This also applies to the European Union, where the number of employees in SMEs is even higher at 65.2% [16].

The economic impact of poor mental health becomes even more apparent when considering the research of Cocker et al. [17], finding that owners and managers of SMEs are 50% less productive when they experience high levels of psychological distress. This is in line with the conclusion of Torrès and Thurik [18] who stated that the health capital of small business owners constitutes the most critical capital for their company: “The smaller the firm the bigger its vulnerability in case of a health problem of the owner, be it physical or mental” (p. 312). Although there is extensive research into workplace mental health, less is known about managers’ stress and mental health at different hierarchical levels in SMEs. Commonly, a differentiation is only made between employees on all levels, and entrepreneurs or managing directors.

Furthermore, managers’ mental health does not only affect themselves, but also their employees: In terms of “emotional contagion”, managers’ emotional state has a significant impact on their employees’ well-being [19–21]. In this context, Bonnesen et al. [22] showed how work-related stress is transferred from managers to their subordinates. Given that employees in SMEs are even closer to their managers’ emotional state, it is assumed that the effect of emotional contagion becomes even more pronounced [20]. Consequently, SME managers’ mental health and its promotion should come into focus, taking the specific working context and challenges of SMEs into account.

Overall, research on mental health promotion in the workplace is extensive, with meta-analyses having established the benefits for organizational stress management trainings for psychological well-being [23–25]. However, less research attention has been given to mental health promotion in SMEs and even less to that of SME managers [26]. To enhance our knowledge on effective health promotion specifically within the context of SMEs, we adapted a well-evaluated group-based stress management training for managers [27] to address the target group of SMEs—a health campaign for SME called KMU-GO. Collecting data of 155 managers in SMEs in Southern Germany, we evaluated the effectiveness of this training with a randomized controlled trial (RCT) design with a waitlist control group. The effectiveness is evaluated based on both psychological indices of mental health and physiological indices. Using an RCT design, we aim to contribute high-quality evidence to the evidence base on workplace health promotion, which is often lacking, but highly needing such rigorous research designs [28]. The stress management training is not restricted to a specific company context and can be offered simultaneously to managers of multiple SMEs within a region. This makes it more cost-efficient for individual SMEs, thus increasing the probability of being able to offer it to their managers, and promoting a local network for SME managers to gain peer support.

### Stress Management in the Context of SMEs

#### Particular challenges of managers in SMEs

In order to gain a better understanding of the target group of managers in SMEs, it is important to take a more differentiated look at their antecedents of work-related stress and the particular challenges. As the SME environment entails different working conditions than those in large companies, it affects SME managers’ roles and responsibilities. Accordingly, the Australian Government initiated a comprehensive research project with 1015 small business owners to gain insight into the unique risk factors faced by small businesses [29]. Most prevalent stressors were the survival of the business, maintaining cashflow, attracting new customers, and worrying about the impact on family. The financial pressure becomes even more apparent when considering that the financial security of employees and their families, as well as that of the owners’ families, depends on the skills and decisions of the owner of SMEs [30]. The risk of bankruptcy or loss of credit is therefore a major stress factor. Owners frequently invest all their assets in their company, so they risk more than just job loss [5]. The fusion with private life becomes apparent in terms of the work-nonwork interface as well. Owners in small companies can find it more challenging to achieve a good balance and separation of these two life domains, given that the workplace is frequently at home and family members contribute to the business [31]. Even if these studies focus on entrepreneurs, it can be assumed that managers at other hierarchical levels in SME also experience a similarly high level of pressure of responsibility. The direct impact of their own actions on the success and thus the continued existence of the company may trigger pressure. This can intensify further through the typically personal and informal relationships with employees [32].

Another highly prevalent group of stressors when owning an SME or being a manager in this context are the work tasks. Managers in SMEs have to fulfill multiple roles and tasks simultaneously. Compared to managers in large organizations, SME managers seem to have more fragmented tasks and they are frequently interrupted, thus having keep switching between multiple roles [33]. At the same time, they have to fulfill many different functions and take on a variety of responsibilities, such as management at all levels as well as technical, human, operational, and conceptual tasks [33]. In this context, Shepherd et al. [34] found a positive association between role stress, such as role conflict or role overload, and the extent of burnout.

Another risk factor is the lack of social support that often accompanies a management position in SMEs [35, 36]. The structures in SMEs commonly do not allow a peer-to-peer exchange on everyday problems or difficult decisions, which can lead to a feeling of isolation and loneliness. This is of specific importance as occupational loneliness increases the vulnerability to burnout in managers of SMEs, in particular when their entrepreneurial orientation is low [37].

As a result of high levels of stress, heavy strain is found in SME managers, but only a minority of these strained managers stay at home and call in sick. The majority appears at work despite being ill, which could be related to the attendance pressure in SMEs, as these managers struggle to transfer their tasks to colleagues to compensate for the loss of productivity caused by their absence due to illness [17].

#### Special challenges of SME in mental health prevention

Although the protection of mental health in the workplace is also enshrined in law in many countries, not all companies are yet able to fully comply with this obligation. In Germany, for example, the percentage of companies conducting a risk assessment of psychological risks in the workplace is significantly lower in smaller companies than in large ones [38]. This seems to be related to a limited knowledge of potential tools and providers as well as limited financial and human resources in SMEs to carry out interventions [39–41]. Well-established instruments such as mental health literacy workshops, employee assistance programs, or stress management trainings, which have already become an integral part of health promotion in large companies, cannot be easily transferred to smaller companies and require adaptation to the particular organizational contexts of SMEs [20]. This lack of suitable health interventions is also due to the fact that specific literature on stressors and antecedents of poor mental health in SMEs is still limited [17]. This particularly affects SME managers who are exposed to unique stressors, while playing a key role in creating healthy workplaces. Furthermore, managers often have limited time resources due to their day-to-day business, making the implementation of health promotion for them considerably more challenging [42].

As aforementioned, only limited research has been conducted into mental health promotion for SME managers. With a systematic review, Erschens et al. [26] identified in the period of 2002 and 2023 only six appropriate intervention studies which have examined stress management trainings for managers in the SME context. The included studies followed very heterogenous approaches and revealed intervention effects, predominantly in cognitive behavioral therapy-based and individualized approaches. These results suggest beneficial effects on managers, but data is limited and rigorous study designs are largely lacking.

### Proposing a mental health campaign tailored to SME managers: KMU-GO

Due to the lack of suitable intervention strategies, well-evaluated approaches that consider the contextual requirements of SMEs are needed. In this regard, managers of SMEs play a particularly important role, as they are exposed to a variety of stress-inducing working conditions, and their health and mental well-being have a much more direct impact on the company and its employees than in large enterprises [18, 20]. Consequently, we designed KMU-GO as an adapted version of an existing stress management training for managers in a large manufacturing company, which was called MAN-GO [27, 43, 44], making it accessible for SME managers. In the randomized controlled trial that evaluated the MAN-GO training, positive intervention effects in terms of lower perceived stress reactivity were found [27]. In addition, seven years post intervention, long-term effects for improved psychological well-being in terms of reduced stress reactivity, symptoms of anxiety and depression, and sleep problems were found [44, 45]. The current study aims to examine the effectiveness of this stress management training with managers in SMEs as well as to replicate the previous results in this specific target context.

Established theoretical models of occupational health, such as the effort-reward imbalance model [46] or the demand-control model [47], explain how work stress affects physical and mental health. Furthermore, based on these models, preventative approaches to managing work stress can be derived. Consequently, these models constitute an important basis for the present stress management training and are integrated there as psychoeducational content in order to sensitize the participating managers to potentially harmful working conditions and to address their everyday experience.

To strengthen self-efficacy in coping with stress and build up resources, this stress management training draws on Lazarus’ *transactional stress model* [1, 48]. This model describes the stress reaction as result of an interaction between person and environment and explains the emergence of stress through the appraisal of demands as threatening and unmanageable. When dealing with stressful experiences, Lazarus and Folkman [48] describe three coping approaches that are addressed with the proposed training: problem-focused coping, emotion-focused coping, and reappraisal as a cognitive coping strategy.

In terms of problem-focused coping, managers are introduced to a variety of tools such as improving time management, dealing with conflicts, or the sustainable implementation of health behaviors. As managers in SMEs are often interrupted in their work processes and have to perform a variety of roles, sensible time management can help to set priorities. Additionally, acquiring strategies for realistic goal-setting can also help to improve health-promoting behaviors, such as active breaks or sleep hygiene, through small-step behavioral changes. This aims to encourage managers to focus on their own individual well-being and may enable to promote healthy behaviors such as a clearer separation between work and private life or avoiding presenteeism.

Strategies to promote emotion-focused coping are important due to the aforementioned existential challenges, as managers in SMEs are often confronted with external influences on which they have little control. These challenges are likely to cause high pressure, without managers being able to change the situation significantly. Furthermore, due to their role overload, managers are faced with frequent social interactions and need to manage multiple business relations. This also includes presenting their company and themselves convincingly and confidently to relevant others like customers, suppliers, or employees. The so-called required “emotion work” draws on good self-regulatory skills, with regulation of emotions playing an important role in the successful completion of work-related tasks [5] as well as managing perceived stress [49]. Emotion-focused coping mechanisms are therefore of great importance in the context of stress management; especially with regard to emotional contagion and the influence on the well-being of their employees. To address these challenges, the training provides various tools such as relaxation and meditation techniques, acceptance strategies and skills to generate positive feelings or manage anger. Owning or managing an SME can feel lonely and isolating. In order to focus on as well as manage such feelings, group discussions were conducted as a crucial training component. These consisted of individual case work and peer-to-peer exchange, enabling the experience of social support. In contrast to an individual coaching approach, the group experience is considered to have a highly significant impact as it provides SME managers—who frequently do not have many peers within their company—with the experience of universality, that is, with the relief that one is not alone with their struggles and shares commonalities with others [50]. Based on the shared experience and joint case work, a social support network is established; an essential resource in managing work-related stress (e.g., [51]). In the qualitative evaluation of MAN-GO, it became apparent that the exchange in the training group played a pivotal role in strengthening social networks and social support [44, 45].

As the reappraisal of a stressful situation both influences the emotional response and enables problem solving, teaching cognitive coping strategies is of fundamental importance. Furthermore, the examination of dysfunctional assumptions and core beliefs may help dealing with exaggerated self-expectations, which are often associated with high levels of stress. With regard to the managers, this may also be inherent within feelings of a high responsibility for employees and their families, which causes a lot of pressure. Teaching cognitive coping strategies supports managers in critically questioning dysfunctional assumptions, adopting a rational and evidence-based perspective, and uncoupling the assessment of their own personal worth from the success of their business.

The underlying idea of this training is to prevent the experience of feeling mentally and physically overwhelmed and to enable the mitigation of stress reactions. Training of coping strategies therefore promotes the belief that one is in control over one’s own behavior, strengthening self-efficacy expectations, which are closely associated with stress reactivity [52]. Accordingly, we expect a positive impact on stress reactivity as a result of the training. As work stress increases the risk of depressive disorders [53], coping with stress may reduce the risk of such mental health conditions. Therefore, we expect a positive impact of the training not only of perceived stress reactivity, but also on stress-related psychiatric pathology like depression and anxiety. Therefore, we propose the following hypotheses:

- *Hypothesis 1*: Perceived stress reactivity will be lower in the intervention group in comparison to the waitlist control group at T1 (Hypothesis 1a) and at T2 (Hypothesis 1b).
- *Hypothesis 2*: Anxiety symptoms will be lower in the intervention group in comparison to the waitlist control group at T1 (Hypothesis 2a) and at T2 (Hypothesis 2b).
- *Hypothesis 3*: Depressive symptoms will be lower in the intervention group in comparison to the waitlist control group at T1 (Hypothesis 3a) and at T2 (Hypothesis 3b).

Based on the model of allostatic load, prolonged exposure to stressors results in the physiological system’s inability to maintain its adaptive allostatis, thus reducing stress coping capacity [11]. The allostatic load—the “chronic wear and tear” [11] of one’s physiological system—can be measured in various physiological stress indices [54]. Consequently, by training SME managers’ stress coping abilities, we expect an improvement in their physiological stress reactions. More specifically, we evaluate changes from baseline to post-training in hair cortisol concentration, which indicates hypothalamic-pituitary-adrenal axis activity [55, 56], as well as in salivary alpha-amylase activity, reflecting basal activity of the sympathetic nervous system [57, 58]. Consequently, we propose the following hypotheses:

- *Hypothesis 4*: Hair cortisol concentration will be lower in the intervention group in comparison to the waitlist control group at T2.
- *Hypothesis 5*: Parameters reflecting salivary alpha-amylase activity will be more favorable in the intervention group in comparison to the waitlist control group at T2, that is, the amylase awakening response will be higher (Hypothesis 5a), whereas the diurnal slope (Hypothesis 5b) and area under the curve in the morning (Hypothesis 5c) and throughout the day (Hypothesis 5d) will be lower.

## Method

To evaluate the KMU-GO intervention, we conducted a study with a randomized 2 × 3-mixed design: factor 1 (group): intervention group or waitlist control group; factor 2 (point in time): baseline, 6 months and 12 months later. A waitlist control group was chosen because this study represents the first evaluation of this intervention in the SME context. This design allowed us to establish evidence of intervention’s effectiveness while ensuring that all participants ultimately received the intervention, which supported both ethical considerations and recruitment feasibility in the SME context. More detail on the study can be found in the study protocol [59]. In line with the study protocol, three further studies evaluate the intervention’s effectiveness in terms of managers’ occupational self-efficacy (Rebecca Erschens C, Schröpel SH, Adam S, Hofmann M, Diebig S, Gritzka et al: Strengthening occupational self-efficacy in SME leaders: results from a randomised controlled trial of a multicomponent training, Under review), its cost effectiveness [60], as well as regarding the participants’ direct employees’ well-being [61].

### Participants and procedure

The target sample consisted of managers in small and medium-sized enterprises (i.e., enterprises with up to 500 employees) in Southern Germany. Interested SME managers had to be between 18 and 64 years old and should not be planning to retire within the next year. It was required that they have managerial responsibility for at least one employee. Furthermore, they should have sufficient German language skills. Participation was voluntary and written consent was obtained before study inclusion. During the recruitment phase, we worked with local multipliers to spread the study invitation throughout Southwest Germany by email, telephone, and information events. These multipliers played a crucial role, as establishing direct contact with SMEs—unlike with large enterprises—is virtually impossible due to their large number. The multipliers included, for example, a health insurance provider and institutions of economic self-governance (e.g., the German *Offensive Mittelstand*, that is, SME offensive). Their networks were utilized to disseminate information about the training, as their communication channels and contact databases were essential in reaching the target group. After written consent to participate in the study, participants were randomly assigned to the intervention group or the waitlist control group. Randomization was conducted by a software program which randomly generated binary numbers for the participants which marked their group assignment.

**Fig. 1:**
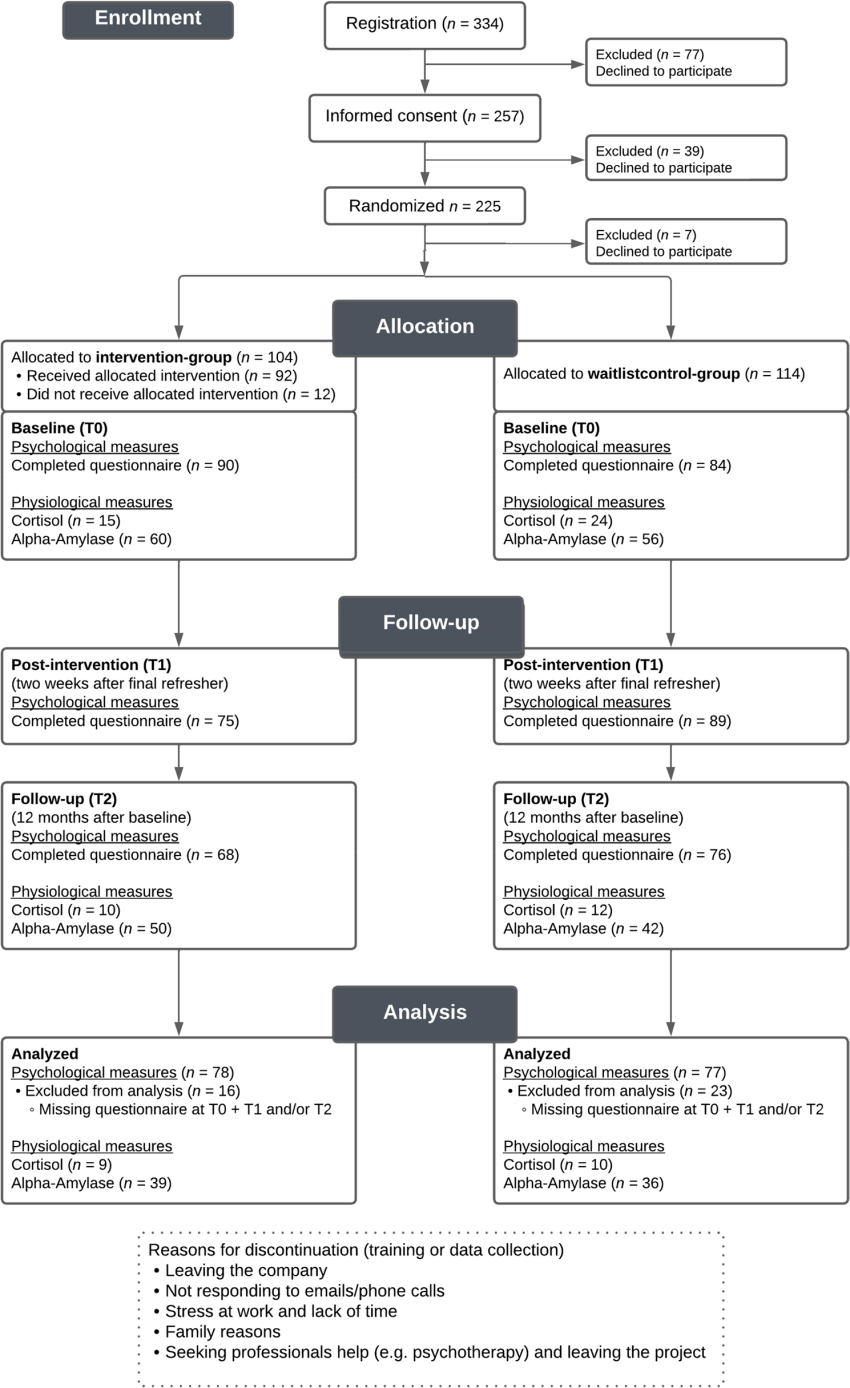
CONSORT flowchart of participants

Participants were sent the link to the online questionnaire two weeks before the training sessions for the intervention group took place (T0, baseline). The baseline questionnaire took about 25 min to complete. The link to the second online questionnaire was sent two weeks after the intervention group had their second refresher session (T1). The link to the third online questionnaire followed another six months later, that is, 12 months after baseline (T2). Adherence to the study was maintained through reminders by e-mail, telephone, and postcard. The physiological measures took place at T0 and T2. After completing the final measurement point, participants in the waitlist control group were provided with a choice of dates for their training sessions. Fig. 1 shows the CONSORT participant flow diagram.

The study started in 2021, during the ongoing COVID-19 pandemic. The baseline assessment and training sessions for the intervention groups were conducted between summer 2021 and winter 2022. These sessions took place in person, in compliance with hygiene regulations, including mandatory mask-wearing, physical distancing, and proof of a negative COVID-19 test. The waitlist control group was implemented between summer 2022 and winter 2023. During this period, in-person meetings remained feasible under continued mask and testing requirements.

The final sample consisted of *N* = 155 managers that completed the baseline measures (T0) as well as at least one of the follow-up measures (i.e., T1 and/or T2). This corresponded to the required sample size previously determined through power analysis [59]. The participating managers were predominantly male (72.3%) and had a university or doctoral degree (or an equivalent occupational degree; 73.5%). Average age was *M* = 45.4 years (*SD* = 9.0), ranging from 26 to 61 years. The participants worked on average *M* = 44.5 h per week (*SD* = 10.0) and had personnel responsibility over *M* = 24.7 employees (*SD* = 65.3). The managerial level was not formally measured, but based on the training groups, managers from all levels participated, from line managers to senior management and owners. Most participants were either married or lived in a committed relationship (87.8%). On average, participants had *M* = 1.3 children (*SD* = 1.2) that lived with them or were dependent on them. Regarding these demographic variables, no significant differences were found between the participants in the intervention group and those in the waitlist control group.^1^

### Intervention

The KMU-GO stress management training is based on a former evaluated stress prevention program for managers in a large company called MAN-GO [27]. To gain deeper insights into the target group’s needs and ensure that organizational aspects were considered in the study design, we engaged with key stakeholders beforehand by conducting semi-structured interviews (*N* = 16). An important goal of these interviews was to ensure optimal accessibility of the training. To achieve this, the training sessions were conducted at two different locations to minimize travel distances. Additionally, the participants were closely involved in the scheduling process. The needs analysis also indicated a significant demand for interactions with other managers. The social exchange was therefore to be strengthened by putting together cross-company and cross-sector training groups. In addition, the training content was conveyed using specific practical examples by incorporating the difficulties and challenges of the interviewees into the training materials. Another emerging need was to strengthen the personal relationship with employees, which was realized by implementing a specific module.

Concerning the didactical frame, the training contained cognitive-behavioral as well as psychodynamic elements and included lectures, discussions, and individual or partner tasks. The training covered the following topics:

- Stress: psychoeducation, effects and consequences of stress, individual stress symptoms.
- Work stress models: gratification model, demand-control-model, organizational fairness.
- Short- and long-term coping strategies on the following levels of response:
  - ◦ Thinking: identification and change of stress-inducing thought patterns and core beliefs.
  - ◦ Emotional response: attention guiding, resource exercises, acceptance strategies, dealing with anger, social support.
  - ◦ Physical response: relaxation and meditation, skills to relieve physical tension, sport, nutrition.
  - ◦ Behaviors: time and break management, Eisenhower matrix to prioritize tasks, conflict management, communication strategies.
- Transformational leadership: staff-oriented leadership, including among others providing an appropriate model, fostering the acceptance of group goals, individualized support.
- Case work: working on individual issues (collegial case advice).

The training sessions were conducted by two health professionals (psychotherapist trained in cognitive-behavioral psychotherapy, medical specialist trained in psychodynamic psychotherapy, or a professional coach) and each training group consisted of up to 12 persons. The training consisted of eight 90-minute sessions held over 1.5 days. Refresher sessions were held after three and six months to further deepen the content and overcome challenges. Each refresher session consisted of two 90-minute sessions.

### Measures

#### Psychological measures

Data on the psychological measures was collected via online questionnaires.

##### Perceived stress reactivity

Perceived stress reactivity was measured with the *Perceived Stress-Reactivity-Scale* (PSRS) by Schlotz et al. [52]. The scale consists of 23 items that cover five sub-scales: reactivity to social assessment (5 items, “If I am wrongly criticized by others .”), reactivity to failure (4 items, e.g., “If I did something wrong .”), reactivity to social conflict (5 items, e.g., “When I argued with other people .“), reactivity to work overload (5 items, e.g., “When I have little time for my work .”), and prolonged reactivity (4 items, e.g., “When I have free time after strenuous work .“). Participants responded to the items by choosing one of three response options that describe their reaction to stressful situations, ranging from 0 to 2. The overall score is calculated by averaging the responses to the 23 items, with a higher score indicating higher perceived stress reactivity. The scale’s internal consistency was good at all three time points (T0: α = .89; T1: α = .89; T2: α = .92).

##### Symptoms of anxiety and depression

Symptoms of anxiety and depression were measured with the *Hospital Anxiety and Depression Scale* (HADS) by Herrmann [62]. The HADS consists of 14 items, asking participants to self-report symptoms of anxiety (seven items; e.g., “Worrying thoughts go through my mind.”) and depression (seven items; e.g., “I still enjoy the things I used to enjoy.”). Participants responded to the items by choosing one of four response options that describe the intensity or frequency of experiencing the described feeling or cognition, ranging from 0 to 3. We calculated an average score for each of the two sub-scales, with higher values indicating a higher level of symptoms. The two sub-scale’s internal consistencies were acceptable to good at all three time points (anxiety: T0: α = 0.76; T1: α = 0.77; T2: α = 0.83; depression: T0: α = 0.80; T1: α = 0.81; T2: α = 0.80).

#### Physiological measures

The physiological measures were collected at baseline (T0) and after 12 months (T2). At these time points, participants came to an appointment where the researchers took hair samples. Prior to these appointments, participants were mailed sample vials and detailed instructions on how to collect and store saliva samples at home, subsequently bringing them to their in-person appointments.

##### Hair cortisol concentration

As a biomarker for the activation of the hypothalamic-pituitary-adrenal axis, hair cortisol concentration was determined from three-centimeter-long hair strands. The length was set at three centimeters to assess the cumulative cortisol release over a period of the past three months as hair have a growth rate of approximately 1 cm/month [63]. To obtain the hair samples, several thin hair bundles were cut as close as possible to posterior vertex region of the head. Hair cortisol concentration was extracted from the collected hair strands following the laboratory protocol described by Feneberg et al. [64]. For cortisol determination, a commercially available cortisol luminescence immunoassay was used (IBL International, a Tecan Group company, Hamburg, Germany). Intra- and inter-assay coefficients of variations were 1.75% and 4.62%, respectively.

##### Salivary alpha-amylase activity

Salivary alpha-amylase activity was used as an indicator of sympathetic nervous system activity and collected via saliva samples. For each data collection point, eight saliva samples were collected over two working days, each day four samples at the same time points: The first sample was taken immediately after awakening, the second 30 min later and the third and fourth samples at 10am and 6pm respectively. Sample collection was performed by the subjects themselves based on detailed instructions. For collection via passive drool, SaliCaps (IBL International, a Tecan Group company, Hamburg, Germany) were used. Subjects were instructed to collect their saliva in the mouth for two minutes and then transfer the accumulated saliva into pre-labelled vials using a straw. Subjects were instructed to keep the samples as cool as possible in their refrigerator or freezer for the period of storage and submit them shortly after to the research team. Subsequently, the samples were stored in the universities’ freezers at -80 degrees Celsius. In order to account for confounding variables, the participants were given a form on which they were asked to note any deviations from the specified times or from the protocol (e.g., eating or intense exercise before sampling). Alpha-amylase activity was measured using a kinetic colorimetric test (for details, see Skoluda et al. [65]) and reagents obtained from DiaSys Diagnostic Systems (Holzheim, Germany). Intra- and inter-assay coefficients of variations were 4.95% and 3.96%, respectively. The following parameters were calculated to evaluate the effectiveness of the intervention: amylase awakening response (AAR), diurnal slope and area under the curve with respect to the ground (AUTCg) in the morning and throughout the whole day.

### Statistical analysis

We applied the intention-to-treat principle, that is, we analyzed the participants according to the group they were originally assigned to [66, 67].

#### Psychological measures

In order to evaluate the effectiveness of the intervention on the psychological measures perceived stress reactivity, anxiety symptoms, and depressive symptoms, we conducted a multilevel analysis of covariance (ANCOVA) with Mplus 8.10 [68] with baseline scores at T0 serving as covariate for the outcome measures at T1 and T2 [69, 70]. Multilevel analysis was conducted to account for the nested structure of data collected at T1 and T2 within participants [71, 72]. Effect sizes for the differences in means between the groups are calculated by dividing the difference in the outcome measures between the intervention group and the waitlist control group by the pooled standard deviation. The effect sizes are interpreted as follows: 0.80 is considered large, 0.50 medium, and 0.20 small [73]. Missing data were handled using full information maximum likelihood (FIML), which incorporates all available observations and provides unbiased estimates under missing-at-random assumptions. Participants with incomplete data (i.e., no data provided at T1 (*n* = 10) or T2 (*n* = 23)) represented a smaller proportion of the sample and their outcome measures at baseline did not differ meaningfully from those with complete data (T0-T2; *n* = 122), indicating that incomplete data is unlikely to have biased the analysis.

#### Physiological measures

To evaluate the intervention effect on the assessed biomarkers of stress, we employed repeated measures analysis of variance (ANOVA) regarding hair cortisol concentration and salivary alpha-amylase activity. These approaches considered both within-subject dependencies and allowed us to assess group by time interactions, providing insights into how our intervention influenced biomarkers over time. Before conducting these analyses, certain data preparations were conducted:

##### Hair cortisol concentration

Due to the skewed distributions of cortisol, these values were logarithmically transformed (log naturalis) before analysis.

##### Salivary alpha-amylase activity

Prior to the analysis of the salivary alpha-amylase activity, the extracted values were *z*-standardized and samples deviating more than three standard deviations were excluded from analysis. This led to the identification and exclusion of a total of 26 outlier samples from further analysis.

In salivary alpha-amylase samples, the rate of missing data was 5.21%. These missing data points primarily resulted from participants forgetting to submit individual samples or singular samples becoming compromised during transportation. To address this issue and ensure the completeness of our dataset, we employed the technique of multiple imputation as a robust and statistically sound method for handling missing data [74]. Multiple imputation is a widely accepted approach that generates multiple plausible imputed datasets to replace missing values, allowing for more accurate statistical analysis [75]. Five imputed datasets were used to ensure robustness. Data points that had been identified as outliers were excluded before multiple imputation was applied.

Before calculating the two AUTCg parameters and the diurnal slope, the salivary alpha-amylase activity values were log-transformed (log naturalis) due to the skewed distribution. The amylase awakening response (AAR) was determined by calculating the difference between the measurement taken upon waking and the measurement after 30 min; both values remaining untransformed. The AUTCg for the morning and the entire day were calculated using the trapezoid formula [76]. For AAR and the two AUTCg parameters, we created a mean variable of the two measured days for T0 and T2, with both days providing data [77, 78]. To evaluate the diurnal slopes of salivary alpha-amylase activity, we used a multilevel regression analysis to predict the salivary amylase activity by time point per participant. By employing this analysis, we extracted the diurnal slopes at T0 and T2 for each participant. In doing so, we considered all available time points, with the exception of the second measurement (awakening + 30 min). This exclusion was made to ensure that our analysis captured the dynamic changes in alpha-amylase activity throughout the study period, while also accounting for the potential influence of the awakening response at the immediate post-awakening time point [79, 80]. For the final analyses, we excluded nine samples (T0: 6; T2: 3) due to self-reported non-compliance with the saliva sampling protocol, such as engaging in excessive physical activity before sample collection, as indicated in the questionnaires.

## Results

### Psychological measures

Table 1 presents the sample descriptive statistics, zero-order correlations, and internal consistencies of the psychological measures across the three time points. Comparing the sample psychological measures at baseline, there were no significant differences between the intervention group and the waitlist control group (see Table 2 for the statistical *t*-test values of the baseline comparisons). Intra-class correlations coefficients (ICC1) were calculated to estimate the amount of variance can be attributed to the within-level (i.e., repeated measures per participant) and how much to the between-level (i.e., between participants). For perceived stress reactivity ICC1 = 0.89, for symptoms of anxiety ICC1 = 0.69, and for depressive symptoms ICC1 = 0.79. These intraclass correlations indicate that between 69% and 89% of the total variance in the psychological variables were attributable to between-person differences, whereas between 11% and 31% were attributable to within-person differences. Further analysis revealed no differences between the participants included in the statistical analysis (*n* = 155) and those who dropped out after baseline (*n* = 18) in terms of the psychological measures.^2^

**Fig. 2:**
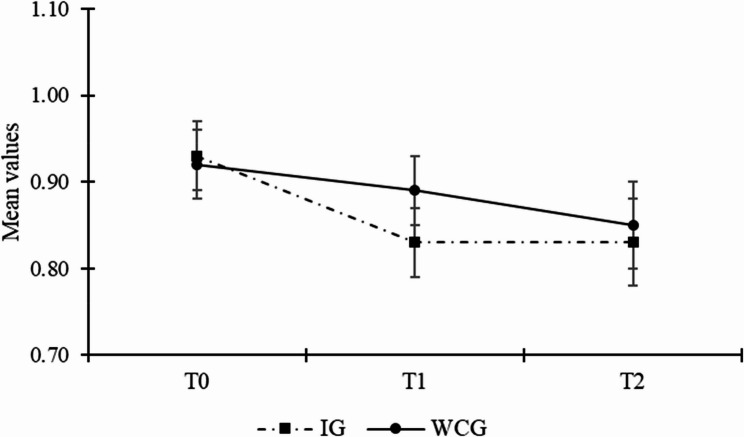
Line graph illustrating the trajectory of perceived stress reactivity per group across the three measurement points. *Note*. Mean values of perceived stress reactivity can range between 0 and 2. Error bars represent standard errors

Table 2 gives an overview of the estimated mean values across time for the two groups, including the test statistics for their comparison. Hypothesis 1 stated that the intervention group would express a lower level of perceived stress reactivity than the waitlist control group after participating in the intervention. At T1, multilevel ANCOVA found a significant mean difference between the intervention group and the waitlist control group (estimated difference = -0.07, *SE* = 0.04, *p* = .048; ES = 0.31), controlling for the baseline level of perceived stress reactivity. More specifically, the intervention group’s estimated perceived stress reactivity was significantly lower than in the waitlist control group (*M*_*IG*_ = 0.82 vs. *M*_*WCG*_ = 0.89), having a small effect size. At T2, there was no significant difference between intervention group and the waitlist control group (estimated difference = -0.04 *SE* = 0.04, *p* = .32; ES = 0.16), controlling for the baseline level of perceived stress reactivity. These findings support Hypothesis 1a, but not Hypothesis 1b. Fig. 2 illustrates the mean values across time for the two groups.

**Table 1:** Sample means, standard deviations, reliability estimates, and intercorrelations among psychological measures

Variable	M	SD	1	2	3	4	5	6	7	8	9
1. Perceived stress reactivity (T0)	0.92	0.35	(0.89)								
2. Perceived stress reactivity (T1)	0.86	0.35	0.79***	(0.89)							
3. Perceived stress reactivity (T2)	0.84	0.37	0.79***	0.89***	(0.92)						
4. Anxiety symptoms (T0)	0.86	0.47	0.61***	0.56***	0.58***	(0.76)					
5. Anxiety symptoms (T1)	0.83	0.47	0.52***	0.64***	0.67***	0.74***	(0.77)				
6. Anxiety symptoms (T2)	0.79	0.52	0.48***	0.51***	0.63***	0.67***	0.70***	(0.83)			
7. Depressive symptoms (T0)	0.61	0.48	0.47***	0.41***	0.37***	0.52***	0.45***	0.35***	(0.80)		
8. Depressive symptoms (T1)	0.60	0.46	0.33***	0.47***	0.44***	0.48***	0.65***	0.50***	0.71***	(0.81)	
9. Depressive symptoms (T2)	0.57	0.45	0.33***	0.38***	0.51***	0.41***	0.51***	0.69***	0.64***	0.79***	(0.80)

Values on perceived stress reactivity can range between 0 and 2; values on anxiety symptoms and depressive symptoms between 0 and 3. Cronbach’s alpha coefficients are reported along the diagonal in parentheses

*N*_*T0*_ = 155; *N*_*T1*_ = 145; *N*_*T2*_ = 132

^*^p < .05

^**^p < .01

^***^p < .001

**Table 2:** Means and standard deviations per group with results of mean comparisons a

Variable	IG		WCG		
	*M*	*SD*	*M*	*SD*	Mean comparison
Perceived stress reactivity					
Baseline (T0)	0.92	0.34	0.92	0.35	*t*(153) = 0.10, *p* = .92, *d* = 0.02
Post-intervention (T1)	0.82	0.22	0.89	0.22	DIFF = -0.07, *SE* = 0.04, *p* = .048; ES = 0.31
Follow-up (T2)	0.83	0.24	0.87	0.25	DIFF = -0.04, *SE* = 0.04, *p* = .32; ES = 0.16
Anxiety symptoms					
Baseline (T0)	0.83	0.44	0.89	0.50	*t*(153) = -0.86, *p* = .39, *d* = 0.14
Post-intervention (T1)	0.76	0.31	0.87	0.31	DIFF = -0.10, *SE* = 0.05, *p* = .04; ES = 0.33
Follow-up (T2)	0.78	0.43	0.83	0.43	DIFF = -0.05, *SE* = 0.07, *p* = .47; ES = 0.12
Depressive symptoms					
Baseline (T0)	0.64	0.45	0.58	0.51	*t*(153) = 0.76, *p* = .45, *d* = 0.12
Post-intervention (T1)	0.51	0.30	0.70	0.30	DIFF = -0.19, *SE* = 0.05, *p* < .001; ES = 0.63
Follow-up (T2)	0.54	0.37	0.63	0.37	DIFF = -0.08, *SE* = 0.06, *p* = .16; ES = 0.23

Values on perceived stress reactivity can range between 0 and 2; values on anxiety symptoms and depressive symptoms between 0 and 3

*IG* Intervention group, *WCG* Waitlist control group, *DIFF* Difference in estimated means, *ES* Effect size

^a^T0 measures represent sample means and standard deviations, whereas T1 and T2 measures represent marginal means and standard deviations.

*N *= 155; N_IG_ = 78; N_WCG_ = 77

**Fig. 3:**
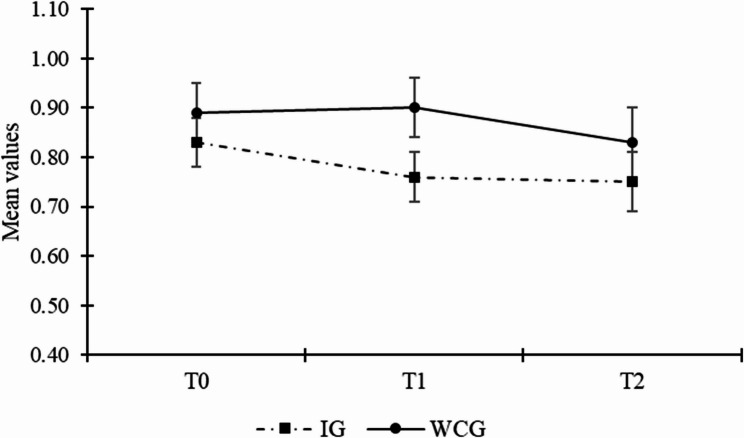
Line graph illustrating the trajectory of anxiety symptoms per group across the three measurement points. *Note*. Mean values of anxiety symptoms can range between 0 and 3. Error bars represent standard errors

With Hypothesis 2, we proposed that the intervention group would express fewer anxiety symptoms than the waitlist control group after participating in the intervention. At T1, multilevel ANCOVA found a significant mean difference between the intervention group and the waitlist control group (estimated difference = -0.10, *SE* = 0.05, *p* = .04; ES = 0.33), controlling for anxiety symptoms at baseline. The intervention group expressed fewer symptoms of anxiety than the waitlist control group (*M*_*IG*_ = 0.76 vs. *M*_*WCG*_ = 0.87) and the effect size for that group difference can be considered small. At T2, we did not find a significant difference between the intervention group and the waitlist control group (estimated difference = -0.05 *SE* = 0.07, *p* = .47; ES = 0.12), controlling for the baseline level of anxiety symptoms. Accordingly, Hypothesis 2a was supported, whereas Hypothesis 2b was not. Fig. 3 illustrates the mean values across time for the two groups.

**Fig. 4:**
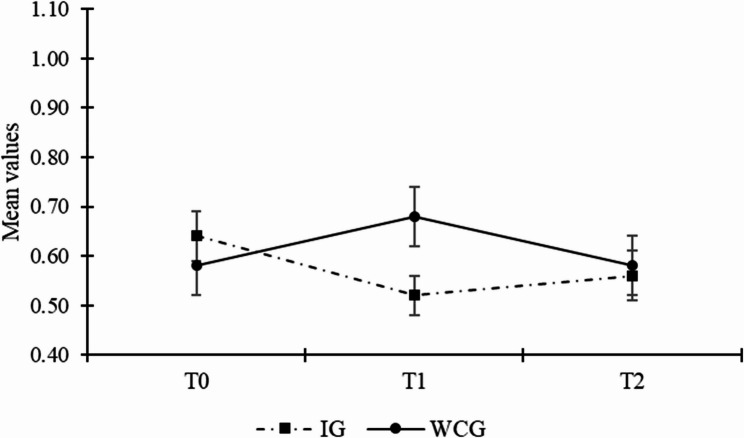
Line graph illustrating the trajectory of depressive symptoms per group across the three measurement points. *Note*. Mean values of depressive symptoms can range between 0 and 3. Error bars represent standard errors

Finally, Hypothesis 3 stated that the intervention group would express fewer depressive symptoms than the waitlist control group after participating in the intervention. At T1, multilevel ANCOVA found a significant mean difference between the intervention group and the waitlist control group (estimated difference = -0.19, *SE* = 0.05, *p* < .001; ES = 0.63), controlling for depressive symptoms at baseline. The level of depressive symptoms was lower in the intervention group than the waitlist control group (*M*_*IG*_ = 0.51 vs. *M*_*WCG*_ = 0.70), indicating a medium-sized effect. At T2, we did not find a significant difference between intervention group and the waitlist control group (estimated difference = -0.08 *SE* = 0.06, *p* = .16; ES = 0.23), controlling for the baseline level of depressive symptoms. These findings support Hypothesis 3a, but not Hypothesis 3b. Fig. 4 illustrates the mean values across time for the two groups.

### Physiological measures

Table 3 outlines the descriptive statistics for hair cortisol concentration and alpha-amylase activity (i.e., AAR, diurnal slope, morning AUTCg, overall AUTCg) by group at T0 and T2 as well as the relevant test statistics. Due to a significant number of participants not completing the physiological measurements, the sample sizes for these analyses were reduced, including *n* = 9/12 (IG/WCG) for hair cortisol, *n* = 40/35 for alpha-amylase baseline, *n* = 35/33 for diurnal slope, *n* = 34/34 for AUTCg morning, and *n* = 33/32 for AUTCg daytime. Comparing the physiological measures at baseline, there was a significant difference for the morning AUTCg (*t*(55.86) = 2.41, *p* = .02, *d* = 0.58), namely the AUTCg was greater for the intervention group than in the waitlist control group. There were no further significant differences between the intervention group and the waitlist control group (see Table 3 for the statistical *t*-test values of the baseline comparisons).

Hypothesis 4 stated that hair cortisol concentration would be lower in the intervention group than in the waitlist control group, indicating lower level of activation of the hypothalamic-pituitary-adrenal axis, a biomarker of stress. The conducted ANOVA did not find a significant interaction between the group and the time points (*F*(1, 19) = 0.30, *p* = .59, $\:{\eta\:}_{p}^{2}$ = 0.02). Accordingly, participation in the intervention did not have a significant effect on the concentration of hair cortisol, thus Hypothesis 4 was not supported.

With Hypothesis 5, we proposed that alpha-amylase activity would be more favorable in the intervention group in comparison to the waitlist control group, indicating a lower level of sympathetic nervous system activity, another biomarker of stress. Conducted ANOVAs did not find a significant interaction between group and time point regarding amylase awakening response (*F*(1, 73) = 1.77, *p* = .19, η^2^ = 0.02), the diurnal slope (*F*(1, 66) = 0.19, *p* = .67, $\:{\eta\:}_{p}^{2}$ = 0.00), and AUTCg throughout the day (*F*(1, 63) = 2.57, *p* = .11, η^2^ = 0.04). There was a significant interaction between group and time regarding the morning AUTCg (*F*(1, 66) = 6.75, *p* = .01, η^2^ = 0.09). However, given the baseline differences between the two groups, we conducted an ANCOVA to corroborate this finding. The ANCOVA tested the mean differences between the two groups at T2, controlling for baseline values. This analysis did not find a significant mean difference between the groups (*F*(1, 65) = 2.24, *p* = .14, η^2^ = 0.03). Accordingly, we conclude that Hypothesis 5 (i.e., Hypotheses 5a-5d) was not supported as participation in the intervention did not lower activation of the sympathetic nervous system activity.

Given the small sample sizes, we conducted non-parametric analyses to validate the reported findings regarding the physiological measures. To replicate the group comparison while adjusting for baseline values, we computed change scores for each participant (T2 – T0) and compared the distributions between the intervention and waitlist groups using Mann–Whitney U tests. For hair cortisol concentration, no significant differences in chance scores were found (*U* = 66.00, *z* = 0.85, *p* = .42). Similarly, in terms of alpha-amylase activity, no significant group differences were found regarding amylase awakening response (*U* = 613.00, *z* = -0.92, *p* = .36), the diurnal slope (*U* = 604.00, *z* = -0.33, *p* = .75), or AUTCg throughout the day (*U* = 614.00, *z* = 1.48, *p* = .14). These non-parametric approaches corroborate the findings from the repeated measures analysis of variance, supporting the robustness of the null effects. Regarding the group comparison of AUTCg in the morning, the comparison of change scores found a significant group differences (*U* = 743.00, *z* = 2.02, *p* = .04). However, given the inconsistent findings of parametric approaches, we would evaluate this finding with caution.

**Table 3:** Means and standard deviations per group with results of t-tests and analyses of variance a

Variable	IG		WCG		
	*M*	*SD*	*M*	*SD*	Comparisons
Hair cortisol concentration (pg/mg)	*N* = 9		*N* = 12		
Baseline (T0)	5.67	5.59	3.98	1.39	*t*(19) = 0.81, *p* = .43, *d* = 0.36
Follow-up (T2)	6.27	7.22	6.81	7.22	*F*(1, 19) = 0.30, *p* = .59,$\:{\eta\:}_{p}^{2}$= 0.02
Alpha-amylase – AR (U/ml)	*N* = 40		*N* = 35		
Baseline (T0)	89.35	92.87	78.23	88.10	*t*(73) = 0.53, *p* = .60, *d* = 0.12
Follow-up (T2)	107.52	99.81	65.23	84.80	*F*(1, 73) = 1.77, *p* = .19,$\:{\eta\:}_{p}^{2}$= 0.02
Alpha-amylase – Diurnal Slope (U/ml)	*N* = 35		*N* = 33		
Baseline (T0)	-2.47	7.53	0.19	5.31	*t*(66) = -1.80, *p* = .08, *d* = 0.44
Follow-up (T2)	-2.88	7.08	1.87	5.62	*F*(1, 66) = 0.19, *p* = .67,$\:{\eta\:}_{p}^{2}$= 0.00
Alpha-amylase – AUTCg morning (U/ml)	*N* = 34		*N* = 34		
Baseline (T0)	395.68	234.12	284.59	184.30	*t*(55.86) = 2.41, *p* = .02, *d* = 0.58
Follow-up (T2)	352.15	218.37	335.55	192.64	*F*(1, 66) = 6.75, *p* = .01,$\:{\eta\:}_{p}^{2}$= 0.09
Alpha-amylase – AUTCg daytime (U/ml)	*N* = 33		*N* = 32		
Baseline (T0)	1149.43	671.88	885.20	483.68	*t*(63) = 1.21, *p* = .23, *d* = 0.30
Follow-up (T2)	1019.32	559.14	965.97	413.66	*F*(1, 63) = 2.57, *p* = .11,$\:{\eta\:}_{p}^{2}$= 0.04

*IG* Intervention group, *WCG* Waitlist control group, *AR* Awakening response, *AUTCg* Area under the curve with respect to the ground

^a^Comparisons partially based on transformed data as described in the section on statistical analysis

## Discussion

The present study evaluated the effectiveness of a stress management training aiming to improve SME managers’ psychological well-being (i.e., perceived stress reactivity, anxiety symptoms, and depressive symptoms) as well as their physiological stress markers (i.e., hair cortisol concentration and salivary alpha-amylase activity). We conducted a randomized controlled trial with SME managers in Southern Germany. Analyzing data of 155 managers, we found significant post-intervention group differences for all psychological measures, with the intervention group showing higher psychological well-being than the waitlist control group. Of the three psychological measures, the strongest effect was observed for depressive symptoms, while the smallest effect was found for perceived stress reactivity. The beneficial mean differences were not found at the follow-up, however, with no significant differences between the groups, indicating that the intervention effects of this study were short-term and did not extend mid-term (i.e., after one year). There were no significant differences between the two groups in terms of the examined biomarkers of stress (i.e., hair cortisol concentration and salivary alpha-amylase activity).

The tested stress management training is based on the transactional stress model and therein proposed coping strategies. Furthermore, the training enabled an experience of universality within a peer group among SME managers, thus facilitating social support. The findings of this study extend the previous knowledge regarding the impact of stress management interventions rooted in the transactional stress model: They demonstrate that providing psychoeducation and training on coping strategies as well as providing coping resources in terms of social support from peers can enhance psychological well-being of SME managers. This target group has previously been under-represented in stress management interventions, lacking accessible stress management support within the SME context [26].

The tested intervention is based on a previously conducted stress management intervention for managers in a large manufacturing company, called MAN-GO [27]. We took the theoretical basis and components of that intervention and made it accessible to regionally distributed SME managers. Like MAN-GO, we found a positive effect on participants’ perceived stress reactivity. In contrast, whereas these improvements were identified 12 months after baseline in MAN-GO, we only found them six months after baseline in this study. Accordingly, it appears that the effects of the present intervention for SME managers is less sustainable than its predecessor addressing managers in large companies. Additionally, parallel to MAN-GO, the present intervention study did not find improvements in symptoms of anxiety and depression 12 months after baseline. We did, however, find improvements six months after baseline. These findings indicate the short-term effectiveness of the present intervention regarding perceived stress reactivity, as well as symptoms of anxiety and depression. These results are also consistent with those of Martin et al. [81], who conducted a CBT-based intervention to promote mental health in SMEs and reported no long-term effect, but rather a short-term reduction in mental stress symptoms. In terms of biomarkers of stress, MAN-GO’s evaluation study examined salivary cortisol concentration and salivary alpha-amylase activity; no significant differences were identified for either set of parameters. This is consistent with the results of the present study. Overall, our findings demonstrate the adaptability of the MAN-GO stress management training for other organizational contexts, as evidenced by its applicability to managers in SMEs.

The beneficial effects for participants’ psychological well-being in this study were found shortly after the training had been finished and not at a subsequent follow-up. Nevertheless, it is noteworthy that the relatively brief initial training duration (i.e., 1.5 days) was sufficient to observe positive training effects, thus underscoring the value of time-efficient stress management approaches. This has implications for future intervention design and for practice. As discussed above, the training’s predecessor, which addressed managers in a large manufacturing company, showed more mid-term beneficial effects for perceived stress reactivity [27] as well as long-term beneficial effects for symptoms of anxiety and depression about seven years after the intervention took place [44, 45]. Accordingly, the beneficial effects of the present intervention might become more apparent over time. Furthermore, these findings could indicate that the chosen intensity and/or duration of the refresher sessions was too low for the specific context to achieve stable long-term effects. In MAN-GO, participants all worked in the same industrial plant, mostly knew each other, and—even more importantly—reported that they kept in touch after the intervention for a long time. In our study, the participants came from different and distantly located SMEs. The peer support network, and thus its benefits for psychological well-being, might have been more challenging to establish and maintain as SME managers were predominantly unfamiliar with each other when meeting in the training context, and keeping in touch after the intervention would have required extra effort from them. Accordingly, the intervention effects could be strengthened and prolonged by giving the SME managers more group contact time to establish their peer network as well as external support through the local business administration and professional SME associations which facilitate the maintenance of the SME manager peer group. Additionally, in SMEs, risks assessments for psychological risk factors as well as occupational health programs are less commonly established than in large organizations [38, 82]. These differences are due to limited financial and human resources [83], as well as a lack of scientific knowledge and specific offers that fit the special needs of SMEs [17]. This could mean that SME managers in our sample might have had less awareness and less experience of mental health prevention in the past, making it more difficult to establish and normalize healthy behavior, requiring more stress management training intensity or duration.

When interpreting the results, it is important to consider the potential impact of the COVID-19 pandemic. The recruitment of managers was considerably delayed, as many were unable to participate due to limited time resources and pandemic-related challenges. This may have affected the sampling process. Nevertheless, a comparison of baseline values between the cohorts of the MAN-GO and KMU-GO study revealed no significant differences. Furthermore, the pandemic might have posed an additional threat to the existence of SMEs in particular, which could have adversely impacted their managers’ psychological well-being. Such effects could have influenced the trajectories of the psychological measures and potentially impeded the detection of more substantial or long-term intervention effects. Consequently, comparability with the results from the MAN-GO study may be limited.

We could not identify group differences in terms of the physiological measures at the 12-month follow-up. One plausible explanation is that training effects, as measured by the psychological indicators, were no longer detectable at this time point, suggesting that any earlier improvements may have attenuated before the physiological assessment, thereby reducing the likelihood of identifying corresponding physiological effects. On the one hand, the chosen physiological measures have been previously applied in the evaluation of stress management interventions. However, they face various challenges as markers of training effectiveness as they are not only affected by stress-related processes, but also situational and behavioral factors. Consequently, disentangling intra-individual fluctuations from inter-individual variability is methodologically demanding, especially in real-life settings, where research rigor has to be balanced with practical feasibility. Accordingly, it is possible that the stress management training could have showed positive physiological effects, which we could not statistically identify. For instance, the sample sizes were much lower for the physiological measures than for the psychological measures. This could mean that the analyses were under-powered and could have identified larger differences between the two groups only. The sample size for hair cortisol concentration was particularly low. It would have required a medium-sized effect to be identifiable with the hair cortisol sample size, while we only found a small effect. Regarding salivary alpha-amylase activity, the effectiveness of the MAN-GO program was evaluated with approximately twice the number of participants, finding a trend towards a significant difference. Conversely, other studies investigating stress management interventions that reported significant effects on salivary alpha-amylase activity had similarly small or even smaller sample sizes, but often distributed the intervention over a longer period of time, incorporating regular sessions throughout. Duchemin et al. [84] and Aguilar-Raab et al. [77], for instance, conducted a mindfulness-based intervention with a comparable amount of intervention hours to our intervention, but distributed their intervention sessions evenly over an eight-week period or three-month period respectively. Both studies identified beneficial effects of their intervention program on salivary alpha-amylase activity.

### Practical implications

The present study indicated the effectiveness and feasibility of a stress management training for SME managers’ psychological well-being. SMEs should integrate such stress management trainings in their managers’ formal training plan, for instance, when someone is taking up a managerial position for the first time. Stress management is a critical skill set and can function as primary intervention in addition to being an effective secondary intervention. As primary intervention, such trainings could raise awareness of stressors at work and related mental health issues through providing psychoeducation. Being aware of these stressors can support managers to engage in coping strategies early on, thus building resilience and maintaining mental health [85–87]. This is not only relevant to the SME managers’ mental health, but also their employees (e.g. [89, 90]).

An important part of our stress management training is the peer support that it provided. SME managers frequently feel lonely and isolated without having direct peers to informally discuss their experiences and challenges with [35, 36]. Given the relevance of social support as a resource and thus as a buffer for stressors [90, 91], it is essential to support SME managers in building this resource. If the SMEs themselves cannot provide this resource internally, it is important that they establish cross-organizational links with other SMEs for their managers to facilitate exchange. Local business administration and professional SME associations have a crucial role in building these cross-organizational networks. Given the prominence of SMEs in many countries [82], providing external support to SMEs is of high economic interest to local business administrations. In Germany, such support could be provided by the local chamber of commerce and industry who provide resources and advice to local businesses and represent these businesses regarding local administration and policies. They could organize regular get-togethers for SME managers for an informal exchange. Furthermore, stress management trainings could be offered via the local business administration rather than being organized by SMEs individually. This would make such trainings more affordable to individual SMEs and therefore more accessible to the target audience. This is highly relevant given that SMEs commonly report to have much lower funds to invest in occupational health programs [39–41]. With regard to the stress management training discussed, its low-threshold feasibility and convincing effects are of great interest to a major health insurance company in Southern Germany as addition to their program of occupational health management.

### Limitations and future research directions

Our intervention evaluation employs a rigorous RCT design which is a methodological strength of our study. Another methodological strength is the recruitment of a considerable number of managers from a variety of SMEs to participate in the RCT and complete the questionnaires, enabling us to test the hypotheses relating to the psychological measures with a suitable sample size as planned in our study protocol [59]. Reaching and recruiting many individuals from the target group was challenging given that there are many SMEs of various sizes, but frequently, they are not centrally organized. In this regard, our recruitment strategy, which utilized local multiplier with strong networks and direct contacts within companies, has proven to be effective. This broad recruitment strategy via various organizational gatekeeps is recommended for future research on this target group.

Despite the methodological strengths, there are a few limitations that have to be considered. Firstly, as mentioned above, the training sessions were conducted during the peak of the COVID-19 pandemic, which was characterized by significant uncertainties and concerns about the future for small businesses. This impeded participant recruitment, as some companies lacked the capacity to participate in the study, potentially leading to sampling bias. Furthermore, the challenges during the pandemic may have contributed to a lack of resources available for a sustainable implementation of training strategies [92, 93].

Secondly, despite using well-established measurement scales, the current findings rely on subjective self-reports. The physiological measures were completed by a much smaller sample size, limiting the interpretation of the results. When collecting physiological data, it is challenging to find a good balance between scientific accuracy, on the one hand, and practical feasibility and non-invasiveness for participants on the other hand [94, 95]. Regarding the hair samples, for instance, many participants were opposed to a few strands of hair being cut from the vertex scalp region for aesthetic reasons. Furthermore, the collection of physiological data at baseline took place during the COVID-19 pandemic. Although necessary hygiene measures were taken to reduce risks of infections, individuals appeared to be in general more hesitant toward physical contacts, avoiding the in-person physiological sample-taking. Another limitation is that the physiological measures were collected only at baseline and 12-month follow-up due to study constraints and to minimize participant burden during an already demanding protocol. Although the absence of T1 physiological data limits the evaluation of the training’s effectiveness shortly after it took place, the two-point assessment still provides meaningful insights and maintains comparability with the MAN-GO study, which used an equivalent measurement schedule. Nonetheless, future studies should endeavor to have parallel psychological and physiological measures at all measurement time points to better understand the timing and durability of intervention effects.

Thirdly, given that the definition of SMEs in our study includes companies with a wide range of employee numbers, it is important to note that the generalizability of the results may be affected, as the challenges faced by managers in very small enterprises are likely to differ from those encountered in larger SMEs. Future studies should further explore this potential heterogeneity. Furthermore, the generalizability of our findings is limited to the context of SMEs in Germany. The participation invitation was circulated openly via SME-related networks and associations and invited anyone that is a manager in SMEs and between 18 and 64 years old. However, although we did not collect explicit data on aspects such as ethnicity, we have to assume that our sample is predominantly white and situated in the context of a Western, industrialized, wealthy, and democratic culture. Furthermore, based on the educational background given in the questionnaire, the sample is highly educated. While higher educational qualifications are more prevalent in management positions within SMEs, this may have introduced a selection bias and limits the transferability of the findings to less privileged groups. Future implementations and related studies should therefore consider targeted outreach and diversified communication channels to ensure that the entire spectrum of SME managers is reached. Finally, the restriction of the training to individuals with sufficient German language skills, could have also limited our sample. Future research could translate the training and test its effectiveness in other cultures and languages to examine the generalizability of our findings.

Additionally, we have to assume a certain level of self-selection into the study. The stress management training was aimed at stressed SME managers who want to learn more about stress management and mental health and who wanted to apply this knowledge to change their behavior and leadership style. This required managers to be in a motivational phase open to change; individuals who are ambivalent to change might have been less likely to engage with such a training to change their engagement with mental health [c.f. 98]. Furthermore, participation also required managers to have the personal resources to engage in such a training to integrate changes into their daily lives. If managers feel too stressed and overloaded at work, they might not have the resources, especially time, so they might be less likely to participate in such trainings; a barrier commonly expressed by managers in SMEs [97]. Given the baseline psychological measures of our sample, indicating moderate stress levels, we might have missed some managers who needed the stress management training the most. Another barrier to participating in stress management trainings is a lack of mental health literacy in the occupational context [98], which also includes a certain stigmatization towards individuals who choose to express mental health issues and sign up to such trainings [99–101].

We found that the proposed stress management training was effective for improving psychological well-being shortly after the training and its refresher sessions took place, but not more long-term. Nevertheless, short-term effects (T1) were observed six months after the 1.5-day training sessions, indicating that the two refresher sessions were able to generate and stabilize positive training effects. Future research should therefore revise this stress management training to provide more sustained benefits for SME managers’ well-being. One possible revision of the training could be the distribution of the training out over a longer period of time by providing refresher sessions for a longer period of time. Sustained health change requires that new behaviors become habitual and are thus maintained over a longer period [96, 102]. For instance, mindfulness-based intervention of similar duration that were spread out over a longer period of time found changes in biomarkers of stress [77, 84]. Furthermore, extending the refresher sessions for a longer period could facilitate a more sustainable peer network among participants as they become more familiar with each other, thus building a more solid trust basis. Maintaining the peer group after the training period could be made a formal part of the refresher sessions. This could include to provide support for participants to establish supportive framework conditions for a sustained peer group (e.g., organizing suitable premises for the peer group via the local business administration or professional SME associations) as well as providing external moderation for the initial peer group meetings until the group establishes their own group structure. Finally, the intervention effects could be strengthened and extended by integrating additional organizational-level measures related to workplace health promotion, such as implementing flexible working schedules such as job sharing or implementing participatory approaches [24, 103]. However, it has to be considered that organizational-level measures are challenging in SMEs and highly conditional on the respective company and its structures. Nevertheless, future adaptations of the training may benefit from coupling individual stress management strategies with feasible organizational measures tailored to the specific SME context, potentially enhancing long-term effectiveness.

Another addition to the training could be to lay the groundwork for the stress management training beforehand. Awareness for mental health issues has been reported to improve participation in interventions aiming to improve mental health at work, whereas lack of such awareness hindered participation (Paterson et al., 2024). Accordingly, interventions focusing on mental health literacy and anti-stigmatization of mental health issues such as awareness campaigns or mental health trainings conducted by peers with lived experience reported could be employed as preparatory steps before the stress management training itself [100, 104, 105].

Another future research direction is the implementation of an active control group. In the present study, we used a waitlist control group. This limits the evaluation of the training’s effectiveness evaluation to a certain extent. On the one hand, improvement in psychological well-being in the intervention group could originate to a certain extent that participants received *something*, regardless of the actual content of the training (i.e., Hawthorne effect). This could also explain why the intervention effects faded at the follow-up. On the other hand, the training effects might have been underestimated by using a waitlist control group. Participants in the waitlist control group expected a training and thus, given they signed up for the training, indicating interest and motivation, anticipated to improve their stress management and their well-being. Accordingly, their well-being might have been improved by these expectations (i.e., expectancy effect), especially at the follow-up measurement point which was shortly before training was provided to the control group participants. In future studies, the training’s effectiveness could be compared to active control groups that receive another type of training. For instance, the present stress management training’s effectiveness could be compared to a more generic stress management training which is not focused on managers, such as a manualized stress management training like mindfulness-based interventions (e.g., [108]). Consequently, it could be tested more rigorously whether the more manager-focused contents are active ingredients for the training’s effectiveness.

## Conclusion

This study provides evidence that a stress management training for managers in SMEs can have a positive effect of perceived stress reactivity as well as symptoms of anxiety and depression. The training was a group-based setting and tailored to the specific context of management in SMEs. To our knowledge, this is the first RCT in Germany to evaluate the effectiveness of a stress management training of this kind addressing managers in SMEs—and just one of a few worldwide [81–109]. Given the findings, this type of training appears to be suitable for this target group. This research contributes to our understanding of how to address stress perception of managers in SMEs, which are sometimes referred to as the “largest employer in many countries”. The training format also proved to be suitable for this specific target group and their needs. Cross-company participation was feasible through its advertising within industry-specific networks. The 1.5-day training sessions with subsequent refresher sessions could easily be integrated into the participants’ working life. Although no significant effects were observed for physiological stress indicators, these measures remain valid and informative. Future studies should aim to include all measurement points parallel to the psychological measures and larger sample sizes to fully capture intervention effects. In order to facilitate the integration of the training content into everyday life and to enhance the sustainability of outcomes, additional refresher sessions and a more sustained peer network implementation should be considered.

## Data Availability

The data are not publicly available as participants did not explicitly consent to their data being shared with researchers outside the research team. Data is only available on reasonable request to the first author. The analysis code is available at the Open Science Framework: https://osf.io/b56jk/files/osfstorage?view_only=85e9a16a9b6542d2a327e1c8b4d75150.

## Footnotes

1 Age: *t*(153) = -0.48, *p* = .63, *d* = -0.08; working hours per week: *t*(150) = -0.67, *p* = .51, *d* = -0.11; personnel responsibility: *t*(153) = -1.09, *p* = .28, *d* = -0.18; number of dependent children: *t*(153) = 0.89, *p* = .37, *d* = 0.14; sex: χ^2^ (1) = 0.35, *p* = .56; education level: χ^2^ (5) = 4.54, *p* = .48; relationship status: χ^2^ (1) = 1.43, *p* = .23.

2 Perceived stress reactivity: *t*(171) = -0.70, *p* = .49, *d* = -0.17; symptoms of anxiety: *t*(171) = -1.40, *p* = .17, *d* = -0.35; depressive symptoms: *t*(171) = -1.15, *p* = .25, *d* = -0.29.

## Abbreviations

AAR: Amylase awakening response
ANCOVA: Analysis of covariance
ANOVA: Analysis of variance
AUTCg: Area under the curve with respect to the ground
IG: Intervention group
HADS: Hospital Anxiety and Depression Scale
ICC1: Intra-class correlations coefficients
KMU-GO: Klein- und mittelständische Unternehmen – Gesundheitsoffensive (Small and medium-sized enterprises – health campaign)
MAN-GO: MAN – Gesundheitsoffensive (MAN – health campaign)
PSRS: Perceived Stress-Reactivity-Scale
RCT: Randomized controlled trial
SME: Small and medium-sized enterprises
WCG: Waitlist control group
